# Time to viral load suppression and its predictors among people living with HIV on antiretroviral therapy in Gebi Resu zone, Afar Region, Ethiopia, 2023

**DOI:** 10.3389/fpubh.2024.1384787

**Published:** 2024-10-28

**Authors:** Anteneh Tefera Chirnet, Ephrem Mannekulih Habtewold, Haji Aman, Elias Bekele Wakwoya, Sewnet Getaye Workie

**Affiliations:** ^1^Department of Public Health, Adama Hospital Medical College, Adama, Ethiopia; ^2^Department of Nursing and Midwifery, College of Health Science, Arsi University, Asella, Ethiopia; ^3^Department of Public Health, School of Public Health, College of Medicine and Health Science, Debre Berhan University, Debre Berhan, Ethiopia

**Keywords:** time to viral load suppression, antiretroviral therapy, predictors, Afar, Ethiopia

## Abstract

**Objective:**

This study aimed to estimate the time to viral load suppression and identify its predictors among HIV patients receiving antiretroviral therapy (ART) in the Gebi Resu zone, Afar Region, Ethiopia, 2023.

**Setting:**

The study was conducted at public health facilities in the Gebi Resu zone of the Afar region.

**Study design:**

This study is a facility-based, retrospective follow-up study.

**Study participants:**

This study included 298 people living with HIV who were receiving ART services at selected health facilities in the Gebi Resu zone. Data were collected by reviewing patient records using a structured checklist. Bivariate and multivariate Cox regression analyses were conducted to assess the relationship between variables and control for confounders.

**Results:**

The incidence rate of viral load suppression was 9.46 per 100 person-months. The median time to viral load suppression was 7.7 months, with an interquartile range of 3.8 months (IQR = 6.47–10.27). Patients at clinical stages 3 and 4 [AHR = 0.67, 95%CI (0.47, 0.96)], those who received cotrimoxazole prophylaxis therapy [AHR = 1.47, 95%CI (1.12, 1.92)], and patients with poor drug adherence [AHR = 0.40, 95%CI (0.18, 0.90)] were significantly associated with time to viral load suppression among people on antiretroviral therapy.

**Conclusion:**

The time to viral load suppression and the median time to viral load suppression among people living with HIV on ART were shorter than those observed in many developing and developed countries. Clinical stage, cotrimoxazole prophylaxis therapy, and drug adherence were significant predictors of viral load suppression.

## Introduction

Antiretroviral therapy (ART) for HIV infection aims to achieve and maintain viral load suppression. This can, in turn, prevent further immune system damage, reduce acquired immune deficiency syndrome (AIDS)-associated morbidity and mortality, restore immune function, and lower the risk of HIV transmission to uninfected individuals. Together, these effects contribute to reducing the overall incidence of HIV (1–4). In Ethiopia, ART became available free of charge in 2005, and antiretroviral treatment was first introduced in 2003 (5). Monitoring the response to ART is critical for determining treatment outcomes in people living with HIV (PLWH). Treatment outcomes are assessed using immunological markers (CD4 T-cell count), World Health Organization (WHO) clinical staging, and routine viral load suppression monitoring. Among these methods, viral load suppression monitoring is considered to be more accurate, timely, and reliable for detecting treatment failure compared to clinical monitoring or CD4 count (immunologic monitoring) (6).

According to WHO guidelines, viral load monitoring should be conducted annually for stable individuals and every 6 and 12 months after beginning ART (7, 8). Viral load suppression is defined by the WHO as the reduction of the virus’s ability to replicate to less than 1,000 copies/ml of plasma after a sufficient duration of ART (9).

The international community has established a global goal to ensure that 95% of all patients undergoing antiretroviral therapy achieve viral suppression by 2025. However, as of 2020, only 66% of the approximately 26 million PLWH on ART had achieved viral load suppression. In 2021, this figure increased to 68% of the 28.7 million PLWH worldwide. Similarly, in Ethiopia, only 72% of HIV-positive patients on ART achieved viral suppression (10, 11).

Multiple factors can lead to an unsuppressed viral load, including patients’ sociodemographic characteristics, treatment adherence, ART regimens, and other clinical factors (4, 12–14).

After a patient has been on ART for at least 6 months, an elevated or unsuppressed viral load could indicate poor adherence to treatment or therapeutic failure due to antiretroviral resistance (7, 15). The probability of CD4 T-cell destruction, the rate at which AIDS advances, and the ease of virus transmission all increase with the number of viral particles in the blood (16). In Ethiopia, among people starting highly active antiretroviral medication, an unsuppressed viral load was found to be a significant predictor of mortality (17). Additionally, studies have shown that 91.5% of new HIV infections were caused by PLWH who were either undiagnosed or did not receive medical attention, that is, those who did not achieve viral load suppression (18).

Despite its importance, only a few studies were conducted in Ethiopia to estimate the time to viral load suppression and its predictors (12, 14, 19, 20), and the time for viral load suppression ranged from a minimum of 3 months in Arba Minch to a maximum of 9 months in Hossana (12, 19).

Given the wide range of findings, conducting a study to assess the median time to viral load suppression in the study area is crucial for improving patient quality of life.

In addition, to the best of our knowledge, very little research has been conducted in the current study region, and there are no data regarding the time to viral load suppression and its predictors for the Afar region, which has been identified as having significantly high clusters of PLWH (21). Therefore, this study was designed to estimate the time to viral load suppression and its predictor in the Gebi Resu zone.

## Methods and materials

### Study design, setting, and population

The facility-based retrospective follow-up study was conducted in ART clinics of public health facilities in Gebi Resu (Zone 3), Afar National Regional State, Ethiopia.

The zone has 11 health centers and 1 general hospital, but only 8 health centers and 1 hospital offer ART services. The study was conducted among PLWH receiving ART services at selected health facilities in the Gebi Resu zone from 11 September 2017 to 10 September 2022. All PLWH who initiated ART for the first time during this period were eligible for inclusion. Since at least two consecutive measurements are required to declare viral load suppression, individuals with less than two consecutive viral load measurements were excluded from the study.

### Sample size, sampling procedure, and measurement

The sample size was calculated using the formulas designed for survival analysis through STATA statistical software, the log-rank test, and the Cox proportional hazard model. The following assumptions were taken into consideration: confidence level = 95%, power = 90%, sample size allocation ratio = 1:1, the hazard ratio of patient’s baseline CD4 level of less than 200 compared to their counterpart 0.683, the hazard ratio of good ART adherence level 2.648, and 1.85 of hazard ratio among patients having opportunistic infection taken from the study conducted in Arba Minch (12) and West Gojam zone (14). Finally, a sample of 298 PLWH was determined after comparing the calculated sample sizes.

Based on the difference in the level of care, we stratified them into hospitals and health centers from a total of nine public health facilities providing ART service in the Gebi Resu zone of the Afar regional state. Only one hospital (Mohammed Akle Referral Hospital) in the zone provided ART service. In addition, three health centers were selected from the seven listed health centers by employing a simple random sampling method. Then, according to the inclusion criteria, eligible PLWH who started ART from 11 September 2017 to 10 September 2022, were identified from the registration book of each selected ART clinic. Finally, study units and selected PLWH receiving ART were randomly selected and allocated proportionally to each selected health facility.

### Operational definition

Viral suppression refers to a viral load less than or equal to 1,000 copies/mL.

The adherence level is classified as good if average adherence is >95% or the patient misses ≤2 from 30 doses and < 3 from 60 doses, fair if the average adherence is 85–94% or the patient misses 3–5 from 30 doses and 3–9 from 60 doses, and poor if the average adherence is <85% or the patient misses ≥6 from 30 doses and > 9 from 60 doses.

Body Mass Index (BMI) is categorized as follows: underweight <18.5 kg/m2, normal weight 18.5–24.99 kg/m2, overweight 25–29.99 kg/m2, and obesity > = 30 kg/m2.

### Data collection procedure and quality assurance

The data for the study were collected by four data collectors who were working on ART service provision, and the collection process was supervised by four trained clinical officers. Data were collected using a structured data abstraction checklist that included sociodemographic variables, clinical factors, and treatment-related variables. The data abstraction format was prepared in the English language. The data were collected through a review of patient medical records, electronic ART databases, and other related registration books (ART registration book, viral load registration book, and HIV-positive tracking registration book) at the selected facilities.

The study’s objectives, purposes, questionnaire, data collection procedures, roles in the evaluation, best practices for high-quality data, confidentiality, and supervision techniques were all covered in a 2-day training session for all data collectors (ART providers) and supervisors (clinical officers). Additionally, the data-gathering tool was pre-tested, and supervisors monitored and verified the data collection procedures. Before processing, the gathered data were stored in a secure location and verified every day for accuracy. After the data were collected, each questionnaire was coded separately.

### Patient and public involvement

Patients were not involved in this study.

### Data analysis

The collected data were coded and entered into Epi-Info version 7.2. These data were then exported to STATA version 16 for processing and analysis. Descriptive statistics such as frequency distribution, percentage, median, and interquartile range were calculated to summarize the characteristics of the study participants. The results of the variables were displayed using frequency tables and graphs. Kaplan–Meier survival curves were used to compare the event times between two or more groups. Observed survival differences were assessed using log-rank tests, with a significance level set at 5%.

Cox proportional-hazard regression was used to identify the predictors of time to viral load suppression among PLWH on ART. The event of interest under this particular objective was viral load suppression after enrolling in ART. The proportional hazard assumption was checked statistically using the global goodness-of-fit test proposed by Schoenfeld. The proportional hazard assumption was fulfilled with a global test value of 0.5661. The assumption was also checked for each predictor with minimum and maximum *p*-values of 0.2350 and 0.9753, respectively. The goodness of fit was checked using the Cox-Snell residual test.

Predictors with *p*-value of less than 0.25 in the bivariate Cox regression analysis were selected as candidates for multiple regression analysis. All predictors were then included in the multiple regression model to identify those independently associated with the outcomes of interest, adjusting for potential confounding variables. Variables with a *p*-value of less than 0.05 were considered as significantly associated with the dependent variable. The strength of the association between dependent and independent variables was expressed as a hazard ratio with a 95% confidence interval.

## Results

### Sociodemographic characteristics of patients

**Figure 1:**
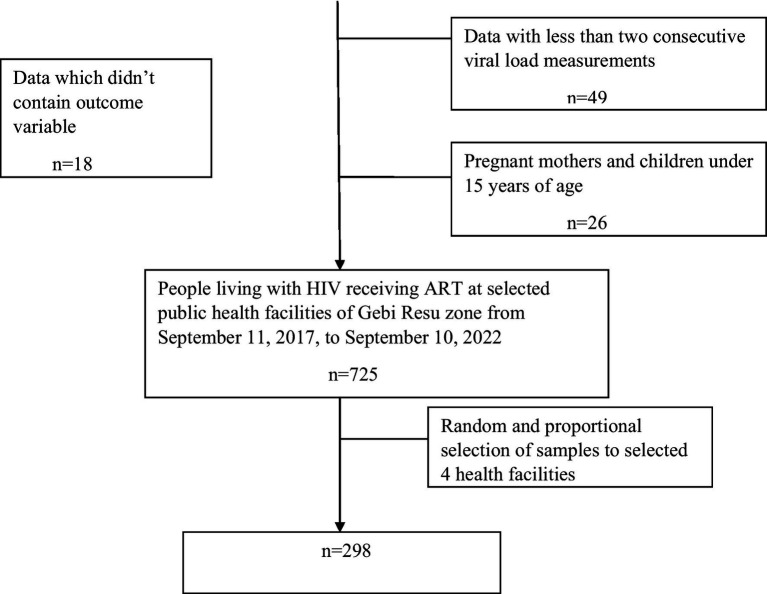
Flow diagram of patient selection to determine the time to viral load suppression and its predictors among PLWH on ART in Gebi Resu zone, Afar region, Ethiopia 2023.

A total of 812 PLWH received ART at public health facilities in the Gebi Resu zone during the data collection period. Of these, 298 participants who fulfilled the inclusion criteria were randomly selected (Figure 1). From a total of 298 PLWH on ART, 204 participants (68.5%) were male individuals, 136 (45.6%) of them were married, 107 participants (35.9%) had no formal education, 205 participants (68.8%) resided in urban areas, 151 participants (50.7%) were Orthodox Christians, 88 participants (29.4%) were private employees, 39 participants (13.1%) were government employees, 27 participants (9.1%) were merchants, and 11 participants (3.7%) were pastoralists. (Table 1).

**Table 1:** Sociodemographic characteristics of PLWH on ART in Gebi Resu zone, Afar Region, Ethiopia, 2023.

Variable categories	Frequency	Percent (%)
Sex		
Female	94	31.5
Male	204	68.5
Age		
15–24	37	12.4
25–34	127	42.6
35–44	93	31.2
> = 45	41	13.8
Marital status		
Single	49	16.4
Married	136	45.6
Divorced	87	29.2
Widowed	26	8.7
Religion		
Orthodox	151	50.7
Muslim	116	38.9
Protestant	31	10.4
Education		
No formal education	107	35.9
Primary	108	36.2
Secondary	52	17.4
Tertiary	31	10.4
Occupation		
Private employee	88	29.5
Government employee	39	13.1
Merchant	27	9.1
Farmer	10	3.4
Pastoralist	11	3.7
Student	46	15.4
Housewife	48	16.1
Not working	29	9.7
Residence		
Urban	205	68.8
Rural	93	31.2

### Treatment-related characteristics of patients

Of the 298 study participants, 109 participants (36.6%) were on a baseline ART regimen of ABC/3TC/NVP or EFV, 254 participants (85.2%) demonstrated good medication adherence, 162 participants (54.4%) received cotrimoxazole prophylaxis therapy (CPT), 195 participants (65.4%) received isoniazid preventive therapy (IPT), and 216 participants (72.5%) disclosed their HIV status to family and friends (Table 2).

**Table 2:** Treatment-related characteristics of PLWH on ART in Gebi Resu zone, Afar Region, Ethiopia, 2023.

Variable categories	Frequency	Percent (%)
Antiretroviral therapy regimen		
ABC/3TC/NVP or EFV	109	36.6
AZT/3TC/NVP or EFV	25	8.4
TDF/3TC/NVP or EFV	164	55.0
Adherence level		
Good	254	85.2
Fair	25	8.4
Poor	19	6.4
Cotrimoxazole preventive therapy		
No	136	45.6
Yes	162	54.4
Isoniazid preventive therapy		
No	103	34.6
Yes	195	65.4
Disclosure status		
Disclosed	216	72.5
Not disclosed	82	27.5

ABC, Abacavir; 3TC, Lamivudine; AZT, Zidovudine; TDF, Tenofovir Disoproxil Fumarate; NVP, Nevirapine; EFV, Efavirenz.

### Clinical characteristics of patients

Of the 298,298 study participants, 208 participants (69.8%) were at WHO clinical stages 1 and 2, 98 participants (32.9%) had previously developed an opportunistic infection, 78 participants (26.2%) had a tuberculosis co-infection, and 213 participants (71.55%) had a baseline CD4 count of greater than or equal to 200 cells/mm.^3^ Additionally, 275 participants (92.3%) had a baseline viral load of less than 1,000 copies/ml, and 208 participants (69.8%) had a baseline body mass index (BMI) greater than 18.5 kg/m^2^ (Table 3).

**Table 3:** Clinical-related characteristics of PLWH on ART in Gebi Resu zone, Afar Region, Ethiopia, 2023.

Variable categories	Frequency	Percent (%)
WHO clinical staging		
Stages 1 and 2	208	69.8
Stages 3 and 4	90	30.2
History of opportunistic infections		
No	200	67.1
Yes	98	32.9
History of tuberculosis		
Negative	220	73.8
Positive	78	26.2
Baseline CD4		
<200	85	28.5
> = 200	213	71.5
Baseline viral load		
<10,000	275	92.3
> = 10,000	23	7.7
Body mass index		
Underweight	90	30.2
Normal	208	69.8
Functional status		
Working	244	81.9
Ambulatory	31	10.4
Bedridden	23	7.7

### Time to viral load suppression and incidence among PLWH on ART

The incidence proportions of people who have achieved first viral load suppression were 83.89% (95% CI (79.25, 87.66)). The total observation time for the 298 patients was 2,644 person-months, and the viral load suppression incidence rate was 9.46 per 100 person-months. The median time to viral load suppression was 7.7 months with an interquartile range of 3.8 months (Q1 = 6.47 and Q3 = 10.27).

**Figure 2:**
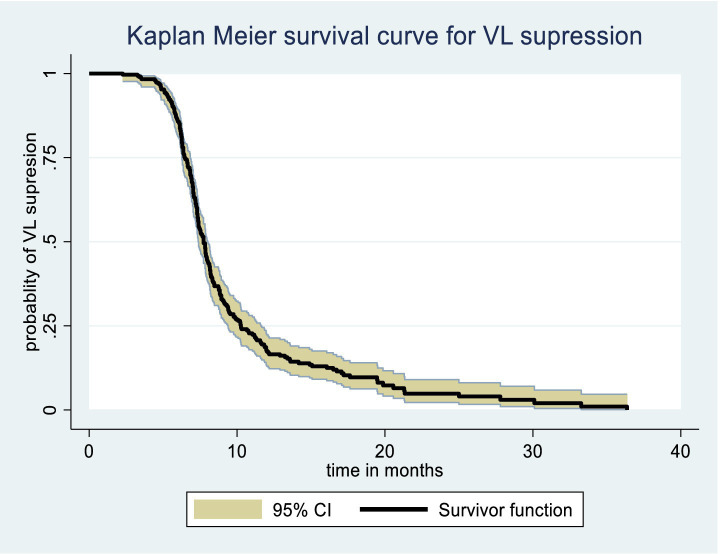
Overall Kaplan Meier survival curve of time to viral load suppression among PLWH on ART in Gebi Resu zone, Afar Region, Ethiopia, 2023.

The Kaplan–Meier survival function graph presents the overall time to viral load suppression, with a median survival time of approximately 8 months, indicating that 50% of PLWH achieve viral load suppression approximately 8 months after initiating ART (Figure 2).

**Figure 3:**
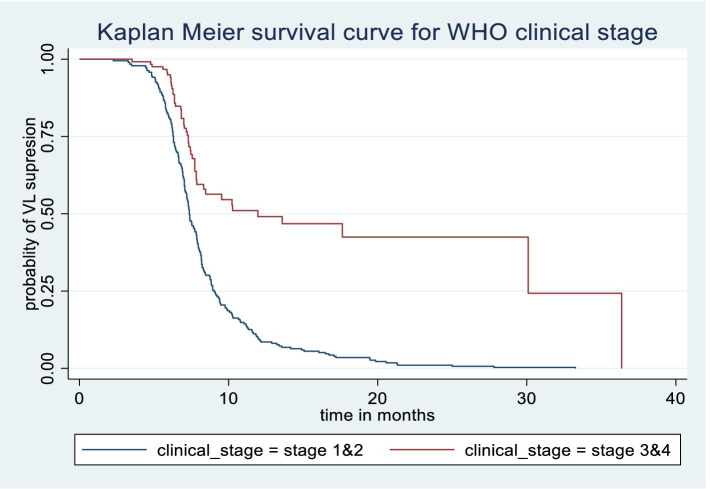
Kaplan Meier survival curve by WHO clinical stage among PLWH on ART in Gebi Resu zone, Afar Region, Ethiopia, 2023. Log-rank test *p*-value = 0.0001.

**Figure 4:**
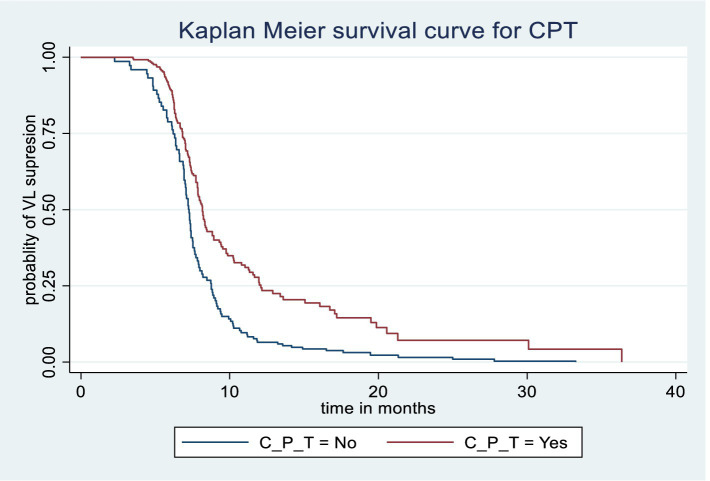
Kaplan Meier survival curve by Cotrimoxazole Prophylaxis Therapy among PLWH on ART in Gebi Resu zone, Afar Region, Ethiopia, 2023. Log-rank test with a *p*-value of 0.0008.

**Figure 5:**
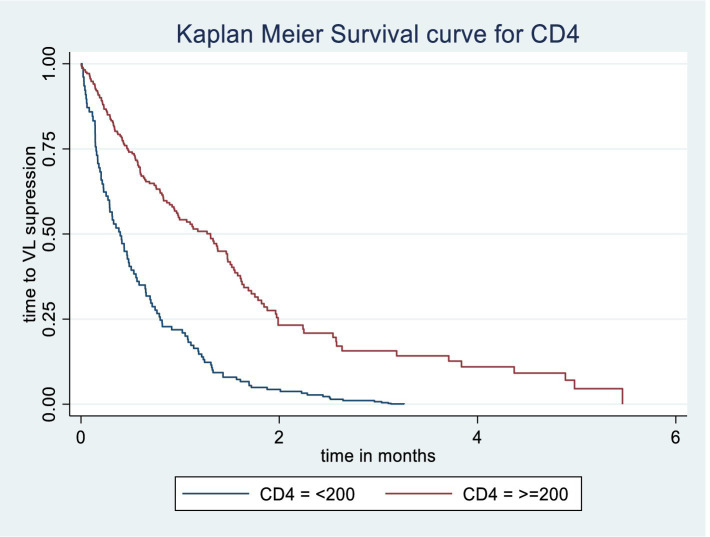
Kaplan Meier survival curve of time to viral load suppression among PLWH on ART by baseline CD4 in Gebi Resu zone, Afar Region, Ethiopia, 2023. Log-rank test with a *p*-value of 0.0143.

**Figure 6:**
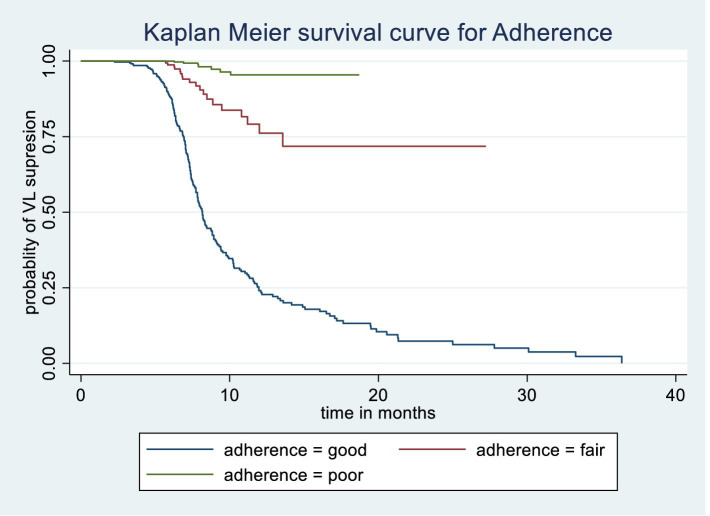
Kaplan Meier survival curve of time to viral load suppression among PLWH on ART by Adherence level in Gebi Resu zone, Afar Region, Ethiopia, 2023. Log-rank test with a *p*-value of 0.0015.

The Kaplan–Meier survival estimate and log-rank test were used to compare the probability of time to viral load suppression across different variable groups. Significant differences in time to viral load suppression were observed between the categories of WHO clinical stage, CPT use, baseline CD4 count, and drug adherence groups (Figures 3–6).

### Predictors of time to viral load suppression among PLWH on ART

Variables with a *p*-value of <0.25 in the bivariate Cox regression analysis, such as sex, age, residence, BMI, WHO clinical stage, adherence, CPT, IPT, baseline CD4, and past opportunistic infection, were included in the multivariate Cox regression model (Table 4).

**Table 4:** Bivariate cox regression analysis of predictors of time to viral load suppression among PLWH on ART in Gebi Resu zone, Afar Region, Ethiopia, 2023.

Variable categories	Outcome		CHR (95%CI)	*p*-value
	VL suppressed	Censored		
Sex				
Female	83 (88.3%)	11 (11.7%)	1	
Male	167 (81.9%)	37 (18.1%)	0.84 (0.64, 1.09)	0.186
Age				
15–24	30 (81.1%)	7 (18.9%)	1	
25–34	111 (87.4%)	16 (12.6%)	1.00 (0.66, 1.51)	0.196
35–44	78 (83.9%)	15 (16.1%)	1.21 (0.79, 1.86)	0.379
> = 45	31 (75.6%)	10 (24.4%)	0.89 (0.53, 1.48)	0.250
Marital status				
Single	42 (85.7%)	7 (14.3%)	1	
Married	116 (85.3%)	20 (14.7%)	0.76 (0.53, 1.09)	0.133
Divorced	71 (81.6%)	16 (18.4%)	0.88 (0.60, 1.29)	0.515
Widowed	21 (80.8%)	5 (19.2%)	0.67 (0.40, 1.14)	0.141
Religion				
Orthodox	130 (86.1%)	21 (13.9%)	1	
Muslim	94 (81.0%)	22 (19.0%)	0.90 (0.69, 1.18)	0.447
Protestant	26 (83.9%)	5 (16.1%)	0.97 (0.63, 1.48)	0.879
Education				
No formal education	89 (83.2%)	18 (16.8%)	1	
Primary	92 (85.2%)	16 (14.8%)	0.93 (0.69, 1.25)	0.625
Secondary	45 (86.5%)	7 (13.5%)	1.00 (0.70, 1.44)	0.991
Tertiary	24 (77.4%)	7 (22.6%)	0.82 (0.52, 1.29)	0.391
Occupation				
private employee	74 (84.1%)	14 (15.9%)	1	
Government employee	31 (79.5%)	8 (20.5%)	0.75 (0.49, 1.14)	0.182
Merchant	21 (77.8%)	6 (22.2%)	1.02 (0.63, 1.66)	0.923
Farmer	6 (60.0%)	4 (40.0%)	0.76 (0.33, 1.74)	0.515
Pastoralist	8 (72.7%)	3 (27.3%)	1.08 (0.52, 2.25)	0.836
Student	41 (89.1%)	5 (10.9%)	1.05 (0.72, 1.54)	0.798
Housewife	43 (89.6%)	5 (10.4%)	1.08 (0.74, 1.58)	0.690
Not working	26 (89.7%)	3 (10.3%)	1.08 (0.69, 1.70)	0.726
Residence				
Urban	188 (91.7%)	17 (8.3%)	1	
Rural	62 (66.7%)	31 (33.3%)	0.83 (0.62, 1.10)	0.102
WHO clinical staging				
Stages 1 and 2	198 (95.2%)	10 (4.8%)	1	
Stages 3 and 4	52 (57.8%)	38 (42.2%)	0.52 (0.38,0.72)**	0.000
Body mass index				
Underweight	66 (73.3%)	24 (26.7%)	1	
Normal	184 (88.5%)	24 (11.5%)	1.43 (0.96, 1.90)	0.097
Functional status				
Working	215 (88.1%)	29 (11.9%)	1	
Ambulatory	21 (67.7%)	10 (32.3%)	0.66 (0.42, 1.05)	0.077
Bedridden	14 (60.9%)	9 (39.1%)	0.63 (0.37, 1.08)	0.095
History of tuberculosis				
Negative	203 (92.3%)	17 (7.7%)	1	
Positive	47 (60.3%)	31 (39.7%)	0.62 (0.02, 1.06)	0.324
Past opportunistic infection				
No	178 (89.0%)	22 (11.0%)	1	
Yes	72 (73.5%)	26 (26.5%)	0.65 (0.50, 0.85)*	0.043
Cotrimoxazole prophylactic therapy				
No	96 (70.6%)	40 (29.4%)	1	
Yes	154 (95.1%)	8 (4.9%)	1.54 (1.19, 1.99)**	0.001
Isoniazid prophylactic therapy				
No	80 (77.7%)	23 (22.3%)	1	
Yes	170 (87.2%)	25 (12.8%)	1.24 (0.95, 1.63)	0.111
Disclosure status				
Disclosed	184 (85.2%)	32 (14.8%)	1	
not disclosed	66 (80.5%)	16 (19.5%)	0.98 (0.74, 1.30)	0.901
Adherence level				
Good	224 (88.2%)	30 (11.8%)	1	
Fair	19 (76.0%)	6 (24.0%)	0.66 (0.41, 1.05)	0.107
Poor	7 (36.8%)	12 (63.2%)	0.25 (0.12, 0.53)**	0.006
Antiretroviral therapy regimen				
ABC/3TC/NVP or EFV	95 (87.2%)	14 (12.8%)	1	
AZT/3TC/NVP or EFV	13 (52.0%)	12 (48.0%)	1.01 (0.56, 1.81)	0.972
TDF/3TC/NVP or EFV	142 (86.6%)	22 (13.4%)	0.18 (0.91, 1.54)	0.211
Baseline CD4				
<200	52 (61.2%)	33 (38.8%)	1	
> = 200	198 (93.0%)	15 (7.0%)	1.22 (0.95, 1.57)*	0.018

^*^Significant variables with a *p*-value of < 0.05.

^**^Significant variables with a *p*-value of < 0.001.

In the multivariate Cox regression model, WHO clinical stage, CPT, and drug adherence were significant predictors of viral load suppression among PLWH with a *p*-value of <0.05 within a 95% confidence interval.

Patients at WHO clinical stages 3 and 4 had lower hazards of achieving viral load suppression by 33% compared to patients at clinical stages 1 and 2 (AHR = 0.67, 95%CI (0.47, 0.96)). Patients who received CPT had 1.47 times higher probability of achieving viral load suppression (AHR = 1.47, 95%CI (1.12, 1.92)). Patients with poor drug adherence had a reduced hazard of viral load suppression by 60% compared to patients with good drug adherence (AHR = 0.40, 95%CI (0.18, 0.90)) (Table 5).

**Table 5:** Bivariate and multivariate Cox regression of predictors of time to viral load suppression among PLWH on ART in Gebi Resu zone, Afar Region, Ethiopia, 2023.

Variable categories	Outcome		CHR (95%CI)	AHR (95%CI)	*p*-value
	VL suppressed (%)	Censored (%)			
Sex					
Female	83 (88.3%)	11 (11.7%)	1	1	
Male	167 (81.9%)	37 (18.1%)	0.84 (0.64, 1.09)	0.85 (0.65, 1.12)	0.315
Age					
15–24	30 (81.1%)	7 (18.9%)	1	1	
25–34	111 (87.4%)	16 (12.6%)	1.00 (0.66, 1.51)	0.69 (0.44, 1.06)	0.279
35–44	78 (83.9%)	15 (16.1%)	1.21 (0.79, 1.86)	0.88 (0.56, 1.39)	0.157
> = 45	31 (75.6%)	10 (24.4%)	0.89 (0.53, 1.48)	0.69 (0.41, 1.19)	0.482
Residence					
Urban	188 (91.7%)	17 (8.3%)	1	1	
Rural	62 (66.7%)	31 (33.3%)	0.83 (0.62, 1.10)	0.87 (0.64, 1.19)	0.361
Body mass index					
Underweight	66 (73.3%)	24 (26.7%)	1	1	
Normal	184 (88.5%)	24 (11.5%)	1.43 (0.96, 1.90)	1.08 (0.79, 1.47)	0.162
WHO clinical staging					
Stages 1 and 2	198 (95.2%)	10 (4.8%)	1	1	
Stages 3 and 4	52 (57.8%)	38 (42.2%)	0.52 (0.38, 0.72)**	0.67 (0.47, 0.96)*	0.026
Functional status					
Working	215 (88.1%)	29 (11.9%)	1	1	
Ambulatory	21 (67.7%)	10 (32.3%)	0.66 (0.42, 1.05)	0.77 (0.47, 1.25)	0.258
Bedridden	14 (60.9%)	9 (39.1%)	0.63 (0.37, 1.08)	0.85 (0.48, 1.50)	0.189
History of opportunistic infections					
No	178 (89.0%)	22 (11.0%)	1	1	
Yes	72 (73.5%)	26 (26.5%)	0.65 (0.50, 0.85)*	0.79 (0.59, 1.05)	0.085
Cotrimoxazole prophylaxis therapy					
No	96 (70.6%)	40 (29.4%)	1	1	
Yes	154 (95.1%)	8 (4.9%)	1.54 (1.19, 1.99)**	1.47 (1.12, 1.92)*	0.013
Isoniazid prophylaxis therapy					
No	80 (77.7%)	23 (22.3%)	1	1	
Yes	170 (87.2%)	25 (12.8%)	1.24 (0.95, 1.63)	1.08 (0.82, 1.43)	0.504
Baseline CD4					
<200	52 (61.2%)	33 (38.8%)	1	1	
> = 200	198 (93.0%)	15 (7.0%)	1.22 (0.95, 1.57)	1.00 (0.76, 1.30)	0.629
Adherence level					
Good	224 (88.2%)	30 (11.8%)	1	1	
Fair	19 (76.0%)	6 (24.0%)	0.66 (0.41, 1.05)	0.79 (0.48, 1.31)	0.187
Poor	7 (36.8%)	12 (63.2%)	0.25 (0.12, 0.53)**	0.41 (0.18, 0.90)*	0.017

^*^Significant variables with a *p*-value of < 0.05.

^**^Significant variables with a *p*-value of < 0.001.

## Discussion

This research aimed to assess the time to viral load suppression and its predictors among PLWH on ART in the Gebi Resu zone, Afar Region, Ethiopia. The incidence proportions of people who have achieved their first viral load suppression were 83.89% (95% CI (79.25, 87.66)). The result aligned with studies conducted in Arba Minch, Ethiopia, and the United States of America (12, 22, 23).

On the other hand, the result was higher than the findings from studies conducted in East Shewa and West Gojjam zones in Ethiopia, Kenya, and Uganda (14, 20, 24, 25). On the other hand, the result was lower than that of a study conducted in Hossana, Ethiopia, Nigeria, and Brazil (19, 26, 27). This discrepancy might be due to the differences between the study areas in sociodemographic, infrastructural, and healthcare provision.

The incidence rate of viral load suppression was 9.46 per 100 person-months. This finding was similar to the results from a study conducted in Hossana, Ethiopia, higher than that of a study conducted in Kenya and lower than that of a study conducted in West Gojjam Zone, Ethiopia (14, 19, 24). The difference might be due to the enhancement in the ART service over time and the difference in socioeconomic status of the participants.

The median time for viral load suppression was 7.7 months (IQR: 6.47–10.27). It aligned with studies conducted in Hossana, Ethiopia, and Italy (19, 28). On the contrary, the median time was longer than studies conducted in Arba Minch and the East Shewa zone in Ethiopia and Switzerland (12, 20, 29). The discrepancy might be due to differences in weather conditions between the study areas. The weather conditions in Afar are very difficult to cope with since it is deserted and sandy (30). It affects the nutritional status of the population, especially PLWH, which would lead to lower antiretroviral adherence and, finally, longer duration of viral load suppression (30, 31). The other reason for the inconsistency might be the socioeconomic differences between the study areas.

The likelihood of achieving viral load suppression among PLWH at WHO clinical stages 3 and 4 was 33% lower than for those at clinical stages 1 and 2. This result aligns with studies conducted in Hossana and Arba Minch in Ethiopia, Kenya, and Nigeria (12, 19, 24, 27). This finding could be explained by the fact that, as HIV progresses to advanced stages, opportunistic infections occur due to immune suppression, which can increase morbidity and mortality rates due to complications (32). Additionally, the suppressed immunity in PLWH at clinical stages 3 and 4, where they develop AIDS, likely contributes to increased viral replication (33).

PLWH who received CPT had a 1.47 times higher likelihood of achieving viral load suppression compared to patients who did not receive CPT. This result is consistent with studies conducted in Arba Minch, Ethiopia (12), Uganda (34), and China (35).

The likely explanation is that CPT reduces susceptibility to bacterial infections, providing an advantage over those not on prophylaxis (36). It is advised that PLWH in clinical stages 3 and 4 take prophylaxis to suppress high viral loads and prevent various opportunistic infections. Additionally, CPT may improve survival rates by reducing malaria and opportunistic infections, thereby strengthening the immune system (37). Studies have also shown that CPT is associated with a slower decline in CD4 levels and a faster reduction in viral load (34, 38).

Patients with poor drug adherence were 60% less likely to achieve viral load suppression compared to those with good drug adherence. The result aligns with the findings from studies conducted in Kenya (24). The observed outcome may be attributed to the critical role of ART in suppressing HIV replication and improving patient outcomes. Good adherence to ART is essential for prolonging the life of PLWH, while poor adherence can have the opposite effect (39–41). Poor adherence diminishes the patient’s immune response, creating opportunities for viral replication, which may also lead to the emergence of drug-resistant strains (42).

The main limitations of the study include the exclusion of important behavioral factors that could directly or indirectly affect the time to viral load suppression, such as alcohol use, smoking, nutritional status, and the patients’ economic conditions. These factors were not accounted for due to the retrospective nature of the study.

## Conclusion

The time to viral load suppression and the median time to viral load suppression among PLWH on ART in the Gebi Resu zone, Afar Region, Ethiopia, were shorter compared to results from both developing and developed countries. The patient’s baseline HIV clinical stage, use of CPT, and adherence to antiretroviral medication were significant predictors of time to viral load suppression in this region in 2023.

Our findings indicate that interventions aimed at improving clinical management, enhancing adherence to ART, and ensuring access to cotrimoxazole prophylaxis for individuals with unsuppressed viral load can significantly contribute to achieving the Joint United Nations Programme on HIV/AIDS (UNAIDS) “last 95%” target in Ethiopia. By focusing on these areas, healthcare providers and policymakers can work toward a more effective response to the HIV epidemic in Ethiopia. This aligns with global initiatives to end AIDS as a public health threat by 2030, reinforcing the need for continued investment in healthcare infrastructure, education, and support systems that empower individuals living with HIV. Further studies should consider assessing behavioral factors that could affect the time to viral load suppression by conducting prospective studies using primary data.

## Data Availability

The raw data supporting the conclusions of this article will be made available by the authors without undue reservation.
